# Supplementary figures and images for: Human PSEN1 Mutant Glia Improve Spatial Learning and Memory in Aged Mice
Source: Cells. 2022 Dec 18;11(24):4116. doi: 10.3390/cells11244116 (PMC9776487; doi:10.3390/cells11244116)

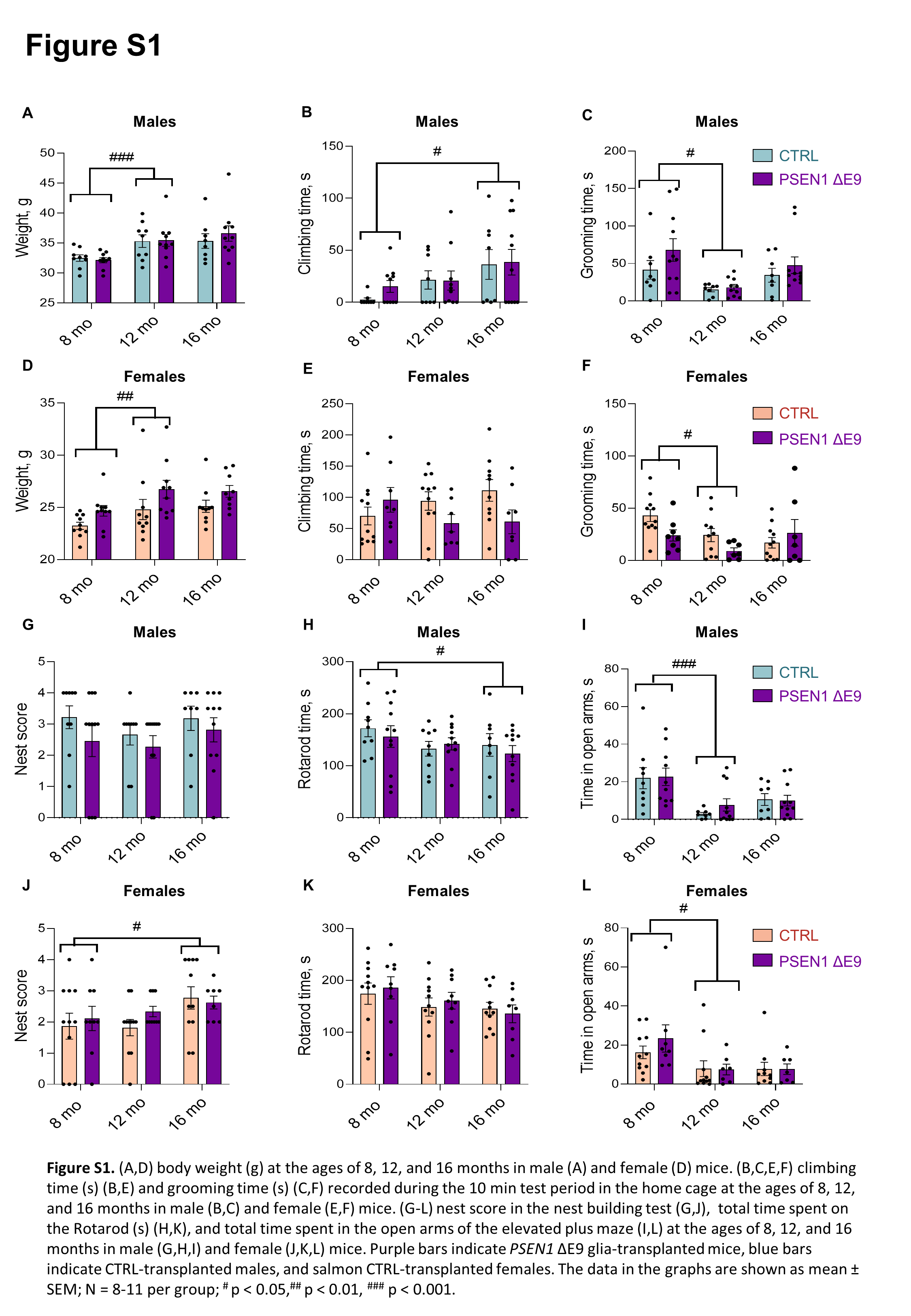

Supplement: Supplementary file 1 [file cells-11-04116-s001.zip › Figure S1.PNG]

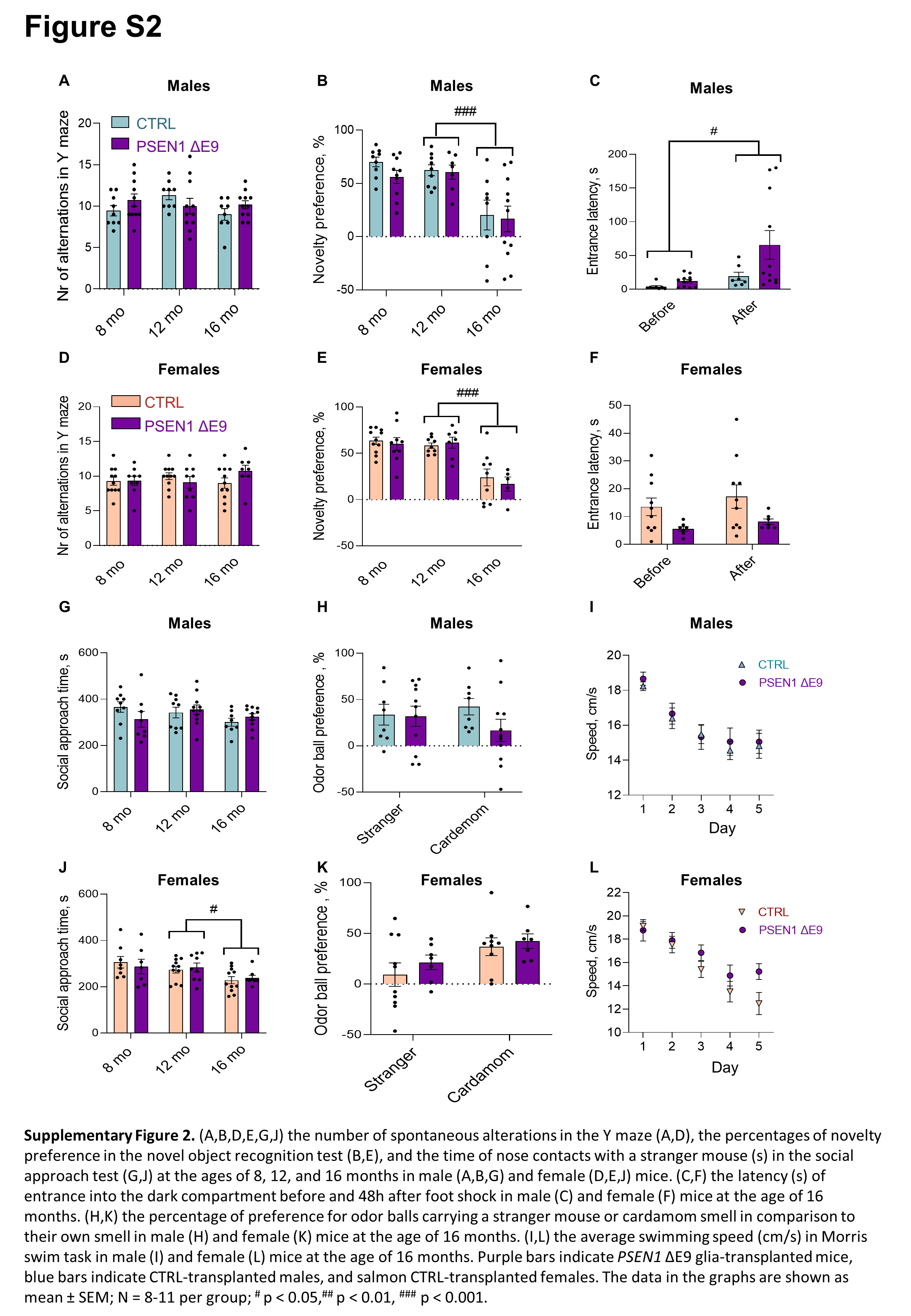

Supplement: Supplementary file 1 [file cells-11-04116-s001.zip › Figure S2.PNG]

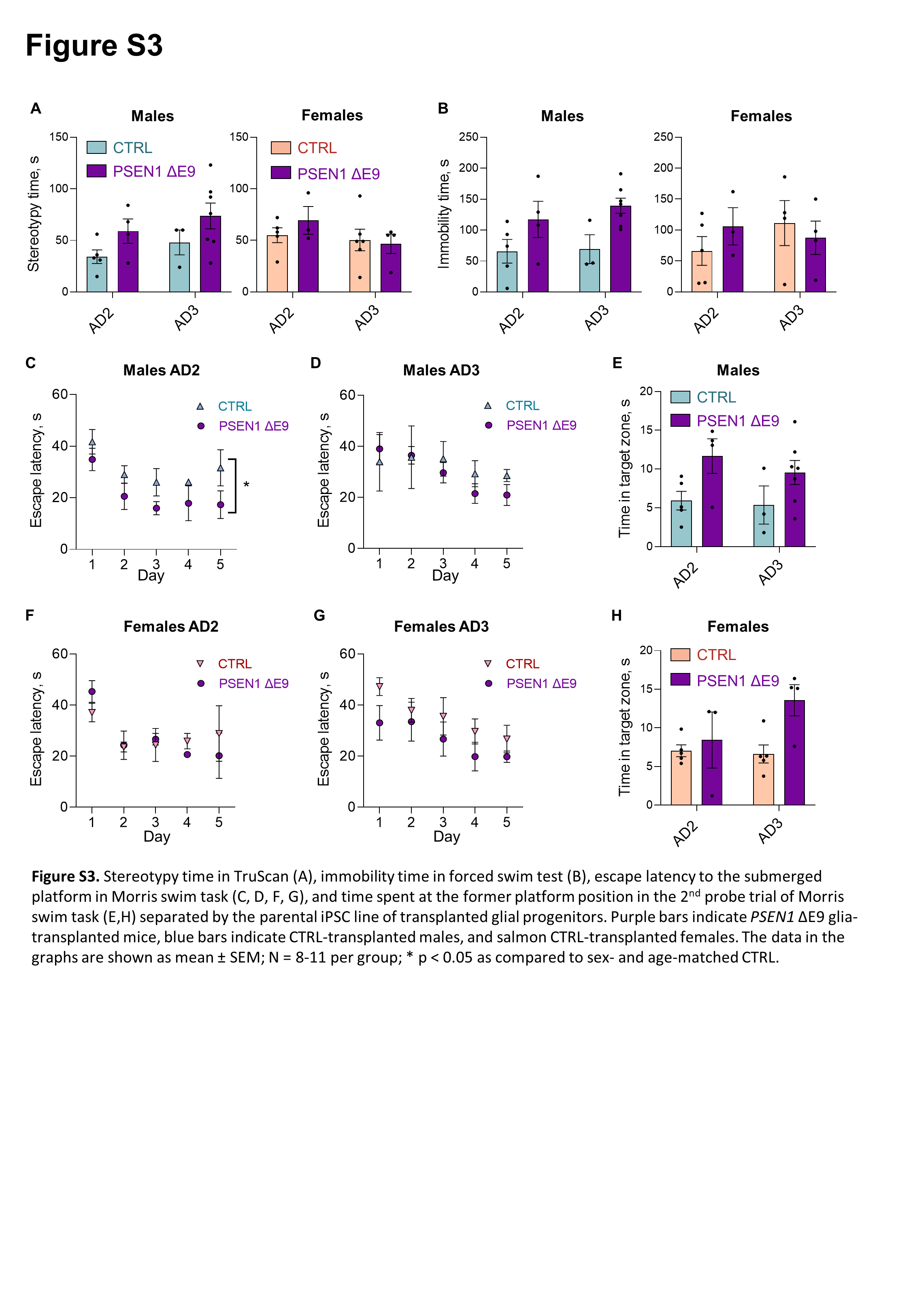

Supplement: Supplementary file 1 [file cells-11-04116-s001.zip › Figure S3.PNG]

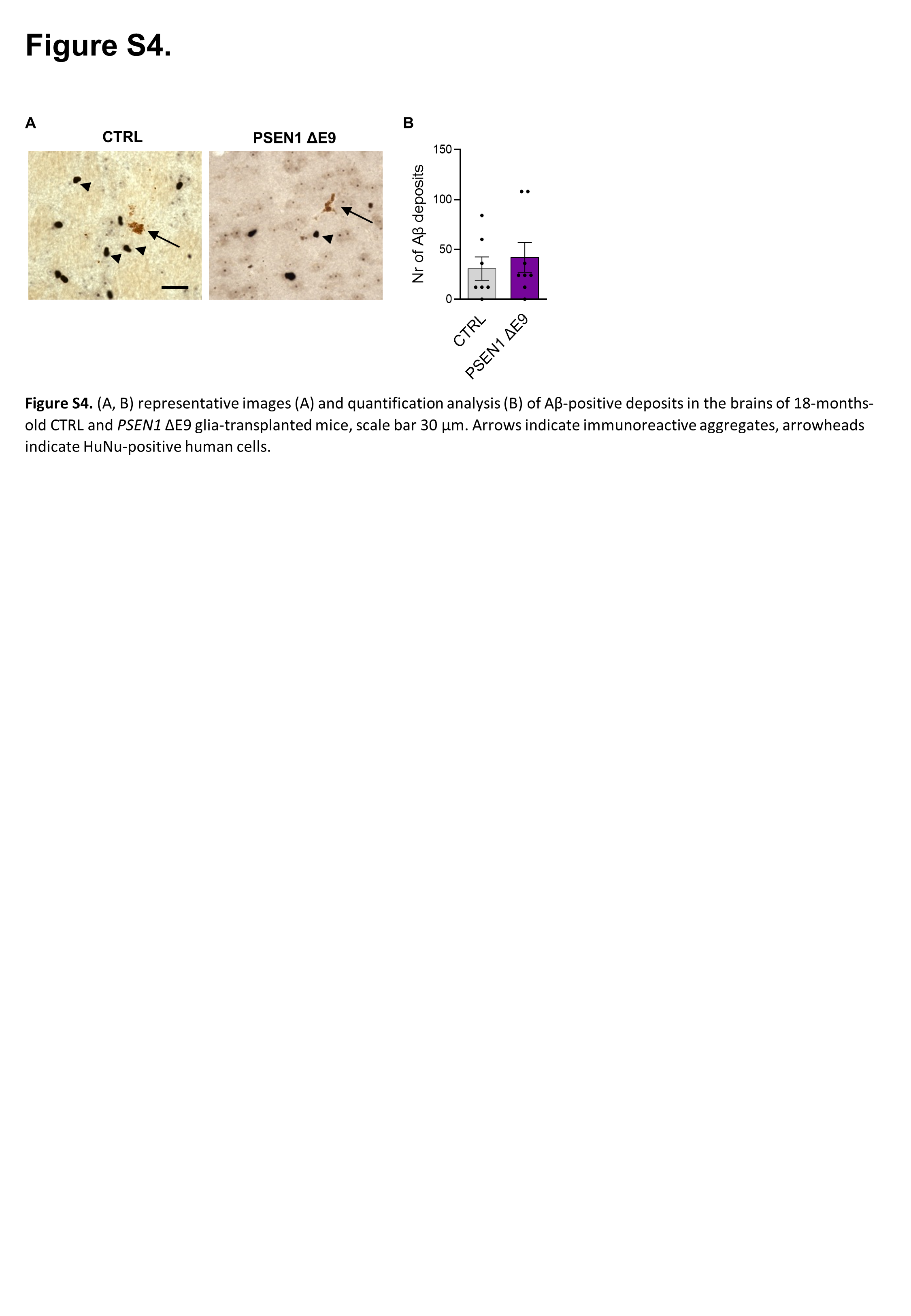

Supplement: Supplementary file 1 [file cells-11-04116-s001.zip › Figure S4.PNG]

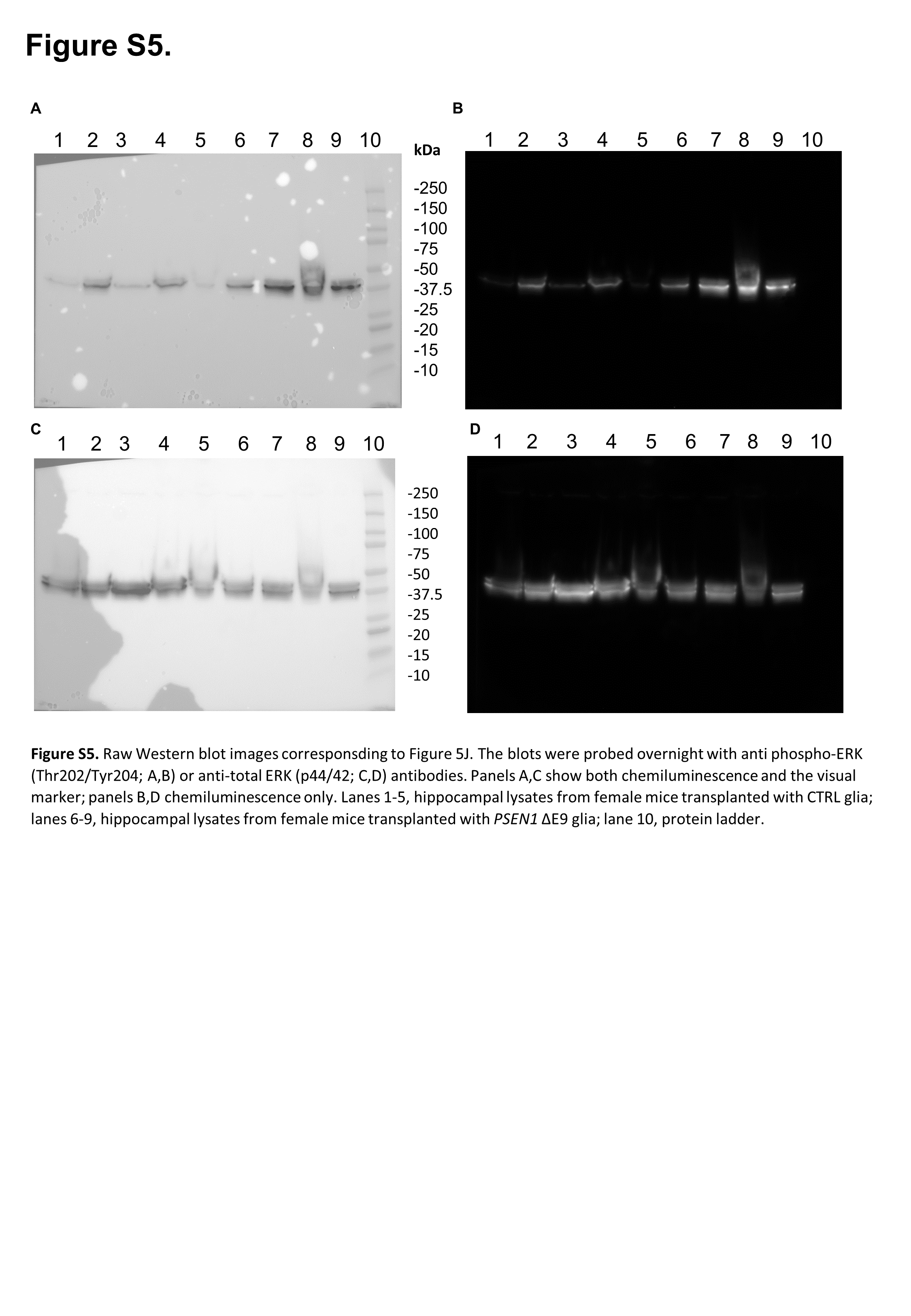

Supplement: Supplementary file 1 [file cells-11-04116-s001.zip › Figure S5.PNG]
